# Effect of Gestational Age on Maternofetal Vascular Function Following Single Maternal Engineered Nanoparticle Exposure

**DOI:** 10.1007/s12012-019-09505-0

**Published:** 2019-02-07

**Authors:** S. B. Fournier, S. Kallontzi, L. Fabris, C. Love, P. A. Stapleton

**Affiliations:** 1 Environmental and Occupational Health Sciences Institute, 170 Frelinghuysen Rd, Piscataway, NJ 08854 USA; 2 Department of Pharmacology and Toxicology, Ernest Mario School of Pharmacy, Rutgers University, 160 Frelinghuysen Rd, Piscataway, NJ 08854 USA; 3 Materials Science and Engineering, Rutgers University, 607 Taylor Rd, Piscataway, NJ 08854 USA; 4 Biology and Environmental Studies, Grinnell College, 1116 Eighth Ave, Grinnell, IA 50112 USA

**Keywords:** Nanomaterials, Nanotoxicology, Wire myography, Uterine artery, Umbilical vein, Fetal aorta, Gestational exposure

## Abstract

Normal pregnancy outcome is accomplished, in part, by rapid and expansive physiological adaptations to the systemic circulation, the extent of which is specific to gestational day (GD) and anatomical location. Pregnancy-related hemodynamic changes in uterine placental blood flow stimulate compensatory vascular signaling and remodeling that begins early and continues throughout gestation. Exposure of the maternal environment to engineered nanomaterials (ENM) during pregnancy has been shown to impact health of the dam, fetus, and adult offspring; however, the consequences of specific temporal (gestational age) and spatial (vascular location) considerations are largely undetermined. We exposed pregnant Sprague–Dawley rats to nano-TiO_2_ aerosols at three critical periods of fetal development (GD 4, 12, and 17) to identify vascular perturbations associated with ENM exposure at these developmental milestones. Vascular reactivity of the maternal thoracic aorta, the uterine artery, the umbilical vein, and the fetal thoracic aorta were evaluated using wire myography on GD 20. While impairments were noted at each level of the maternofetal vascular tree and at each exposure day, our results indicate the greatest effects may be identified within the fetal vasculature (umbilical vein and fetal aorta), wherein effects of a single maternal inhalational exposure to nano-TiO_2_ on GD 4 modified responses to cholinergic, NO, and α-adrenergic signaling.

## Introduction

Gestation is characterized by significant physiological adaptations to accommodate the developing fetus. Normal pregnancy outcome is accomplished, in part, by rapid and expansive physiological adaptations to the systemic circulation, the extent of which is specific to gestational day and anatomic location. Pregnancy-related hemodynamic changes in uterine placental blood flow stimulate compensatory vascular signaling and remodeling that begins early and continues throughout gestation [1]. It is well established that disturbances in the maternal and intrauterine environment during gestation can have a long-term impact on fetal health. Common pregnancy complications, including preeclampsia, and intrauterine growth restriction (IUGR) are of vascular origin. Under circumstances of an adverse intrauterine environment, the developing fetus is vulnerable to oxygen and nutrient deprivation, impairing fetal growth and development.

Unique material attributes emerge at the nanoscale. Nanomaterials are designed and incorporated with increasing frequency in manufacturing processes, commercial products, and biomedical applications, raising the probability of occupational, environmental and therapeutic exposure. To meet growing consumer demands, the annual global production of nanosized titanium dioxide particles (nano-TiO_2_) is projected to reach 2.5 million metric tons by 2025 [2]. The pervasive use of ENM raises concerns regarding their safety and potential risks to human health in all life stages.

Evidence from toxicological literature connects engineered nanomaterial (ENM) exposure to adverse cardiovascular endpoints [3–12]. In non-pregnant animals, ENM inhalation leads to impaired coronary [13] and uterine [14] microvascular dysfunction. This blunted hemodynamic response may be impaired for days even after a single exposure [14]. The rapid implementation of ENM and the broad range of current and potential biomedical applications raise concerns about human exposure to ENM during pregnancy and the subsequent risks to maternal and fetal health.

Within a pregnant population this is concerning as significant growth and development occurs at a rapid pace. Early in gestation, the rat blastocyst implants into the uterine endometrium and initiates trophoblast invasion and placental development. If the uterus is non-receptive, implantation can fail. A complete rat embryo is formed on GD 12.5; with respect to cardiovascular formation, septation begins, atrioventricular bundle begins to form, and vasculogenesis is initiated [15]. Pups transition to the first fetal stage on GD 17, wherein cardiovascular maturity is initiated through septal closures, valvular formation, and continued angiogenesis [15]. Perturbation at each of these stages could severely impair fetal health.

We and others have reported evidence to suggest reproductive and developmental toxicities associated with maternal ENM inhalation or instillation; however, the exposure parameters range widely. Our previous studies have focused on early chronic exposures, initiated on GD 4 through GD 19 [16] or single late exposures initiated on GD 19 [14]. Other groups have developed experimental designs wherein repeated exposures occur during developmental milestones [17–19]. Further, differential vascular responses have been reported after a single pulmonary or intravenous maternal exposure near the end of gestation (GD) [17–22]. While there is little homogeneity in these studies, given each group is focused on the maternal toxicity of a different ENM, there is uniformity in that significant implications are reported in maternal and fetal health outcomes.

Together, the inherent complexities of physiological systems may contribute to the differential tissue responses reported with maternal exposure to ENM during pregnancy. Therefore, the purpose of this study was to identify a potential “critical window” of gestational exposure to ENM on fetal health and provide a macrovascular serial assessment to identify if the greatest vascular dysfunction is of maternal or fetal origin.

## Materials and Methods

### Nanomaterial Characterization

Nano-titanium dioxide (nano-TiO_2_) powder was acquired from Evonik (Aeroxide TiO_2_, Parsippany, NJ). Previous characterization of this powder has determined the composition to be primarily anatase (80%) and rutile (20%) TiO_2_, with a primary particle size of 21 nm and a surface area of 48.08 mg^2^/g. Characterization of these particles was re-confirmed via dynamic light scattering (DLS) techniques using Zetasizer Nano ZS by Malvern. The size of the agglomerated nanoparticles in diH_2_O was measured as 231 ± 21 nm with Non-Invasive Backscatter optics (NIBS) using a 4-mW, 633-nm laser. TiO_2_ powder was dried, sieved, and stored under vacuum prior to aerosolization.

### Animal Model

Timed-pregnant Sprague–Dawley (SD) rats were purchased from Charles River Laboratories (Kingston, NY) and allowed *ad libitum* access to food and water. All animals were randomly assigned to Naïve, Sham, or Exposure groups. Animals in either the Sham or Exposure groups were exposed to filtered air (Sham) or nano-TiO_2_, respectively, at GD 4, 12, or 17. Animals were euthanized on GD 20. All procedures were approved by the Institutional Animal Care and Use Committee of Rutgers University and were executed in accordance with the standards set forth in the “Guide for the Care and Use of Laboratory Animals” of the National Research Council of the National Academies.

### Engineered Nanomaterial Inhalation Exposure

Animal exposures to nano-TiO_2_ were performed using a custom rodent inhalation facility designed for whole-body aerosolized nanomaterial inhalation (IEStechno, Morgantown, WV). The collective exposure system consists of a vibrating fluidized bed, a Venturi vacuum pump, cyclone separator, impactor and mixing device, an animal housing chamber, and real-time monitoring devices with feedback control. Nano-TiO_2_ aerosols were generated via a high-velocity air stream passing through the vibrating fluidized bed and into the Venturi vacuum pump. From there, the aerosols entered the cyclone separator to remove agglomerates greater than 400 nm at an input flow rate of 60 L/min of clean dry air before entering the exposure chamber. Size distribution and relative mass concentration (133.73 ± 1.87 nm) of the aerosols were monitored in real time with a Scanning Mobility Particle Sizer (SMPS, TSI, Shoreview, MN). GD 4, 12, and 17 pregnant rats were placed in the 84-L whole-body exposure chamber for 5 h during a single inhalation exposure. Once a steady-state aerosol concentration was achieved, exposure duration was a single 4-h exposure at a final mass concentration of 9.71 ± 0.22 mg/m^3^ to achieve a daily calculated pulmonary deposition of 15.8 ± 1.2 µg. Animals were removed from the facility when concentrations with the chamber are below 1 mg/m^3^. All rats recovered for at least 24 h prior to experimental procedures. Sham animals were exposed to HEPA filtered air only. For the duration of the inhalation exposure, aerosol concentrations were verified using gravimetric sampling. Aerosols were collected on a 47-nm PTFE membrane filter and an XP2U microbalance (Mettler Toledo, Switzerland) was used to determine filter mass.

### Mean Arterial Pressure (MAP)

Rats were anesthetized with isoflurane gas (5% induction, 3–5% maintenance). The right carotid artery was cannulated to acquire mean arterial pressure (MAP). A BLPR2 pressure transducer (World Precision Instruments, Sarasota, FL) was used in conjunction with a blood pressure monitor (World Precision Instruments, Sarasota, FL) to measure and record MAP.

### Wire Myography

**Fig. 1:**
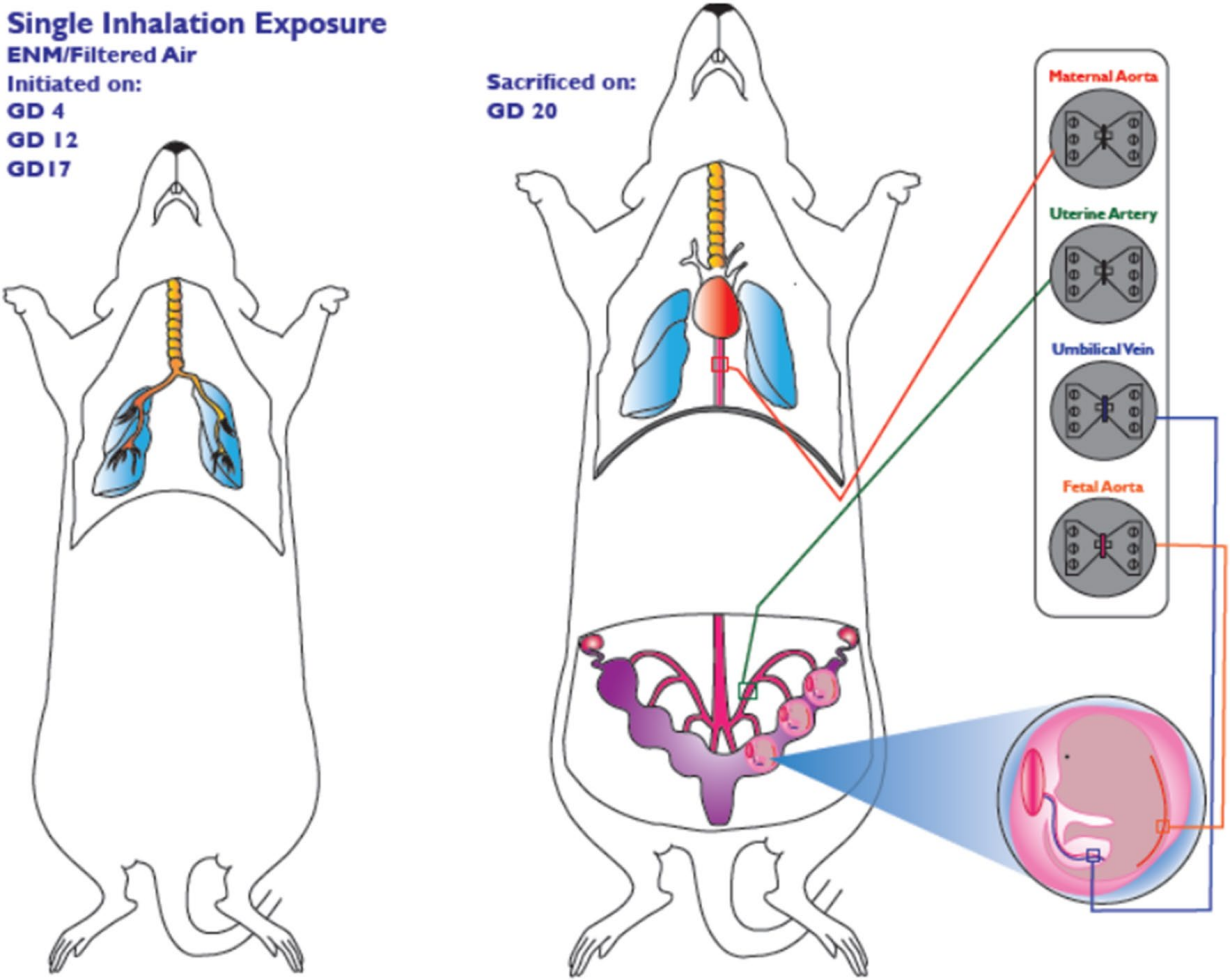
Experimental design schematic. All animals were sacrificed on GD 20

Wire myography (DMT-USA) was used to evaluate vascular reactivity in the maternal thoracic aorta, the uterine artery, the umbilical vein, and the fetal aorta (Fig. 1). Vessels were isolated, excised, and placed in cold physiological salt solution (PSS). Vessel segments (2 mm) were trimmed and transferred to a stainless-steel chamber, submerged in 6-mL chilled PSS (PSS; 130 mM NaCl, 4.7 mM KCl, 1.18 mM KH_2_PO_4_, 1.17 mM MgSO_4_ 7H_2_O, 1.6 mM CaCl_2_, 14.9 mM NaHCO_3_, 0.026 mM EDTA, and 5.5 mM glucose), and mounted on two intraluminal stainless-steel wires (0.04 mm) in a multi-channel wire myograph (620M, DMT, Aarhus, Denmark). Once mounted, vessel chambers were heated slowly to 37 °C and bubbled gently with carbogen (95% O_2_/5% CO_2_). Following 20 min of equilibration, passive length-tension relationships were established for each vessel segment by stepwise stretching of the tissue rings. The optimum resting tension for each vessel segment was calculated based on the La of LaPlace 90% of the internal circumference established at tensions equivalent to 100 mmHg (maternal aorta), 60 mmHg (uterine artery), and 40 mmHg (umbilical vein and fetal aorta). Once normalized, vessel segments were equilibrated for 40 min followed by a brief “wake-up” protocol in which maximum contractile response was determined using high-potassium PSS (KPSS; 74.7 mM NaCl, 60 mM KCl, 1.18 mM KH_2_PO_4_, 1.17 mM MgSO_4_ 7H_2_O, 1.6 mM CaCl_2_, 14.9 mM NaHCO_3_, 0.026 mM EDTA, and 5.5 mM glucose). Vessels were then washed over a period of 20 min with PSS and allowed to relax until initial tension was reached. Contractile responses were determined *via* cumulative additions of 60 µL of phenylephrine (PHE; 1 × 10^−9^ to 1 × 10^−4^ M). Relaxation responses were evaluated *via* cumulative addition of 60 µL methacholine (MCH; 1 × 10^−9^ to 1 × 10^−4^ M) or sodium nitroprusside (SNP; 1 × 10^−9^ to 1 × 10^−4^ M). Responses to chemical agents were randomized.

### Pharmacological Agents for Vascular Studies

Phenylephrine (ACROS Organics, Geel, Belgium) was dissolved in PSS. Sodium nitroprusside (Thermo Fisher Scientific, Waltham, MA) was dissolved in PSS. Methacholine (MP Biomedicals LLC, Santa Ana, CA) was dissolved in PSS.

### Wire Myography Calculations

Maximum tension was defined as the tension developed by the vessel segments with the addition of high-potassium PSS (KPSS; 74.7 mM NaCl, 60 mM KCl, 1.18 mM KH_2_PO_4_, 1.17 mM MgSO_4_ 7H_2_O, 1.6 mM CaCl_2_, 14.9 mM NaHCO_3_, 0.026 mM EDTA, and 5.5 mM glucose). Tension at baseline and with agonists (MCH, SNP, or PHE) was recorded and the percentage of maximum tension generation was calculated using the following formula:

$${\text{Percentage maximum tension }}\left( \% \right)=\left[ {\left( {{\text{Tension with agonists }}\left( {{\text{mN}}} \right) - {\text{Tension at baseline }}\left( {{\text{mN}}} \right)} \right)/{\text{Maximum tension }}\left( {{\text{mN}}} \right)} \right]*{\text{1}}00.$$

### Statistics

Point-to-point differences in the dose–response curves were evaluated using two-way repeated measures analysis of variance (ANOVA) with a Tukey’s *post hoc* analysis when significance was found. The area under the concentration/dose response data of the concentration/dose response curves were determined through non-linear regression. All statistical analyses were completed with GraphPad Prism 7.0 (San Diego, CA, USA). All data are expressed as mean ± SEM and significance is set at *p* ≤ 0.05.

## Results

### TiO_2_ Nanoparticle Characterization

**Fig. 2:**
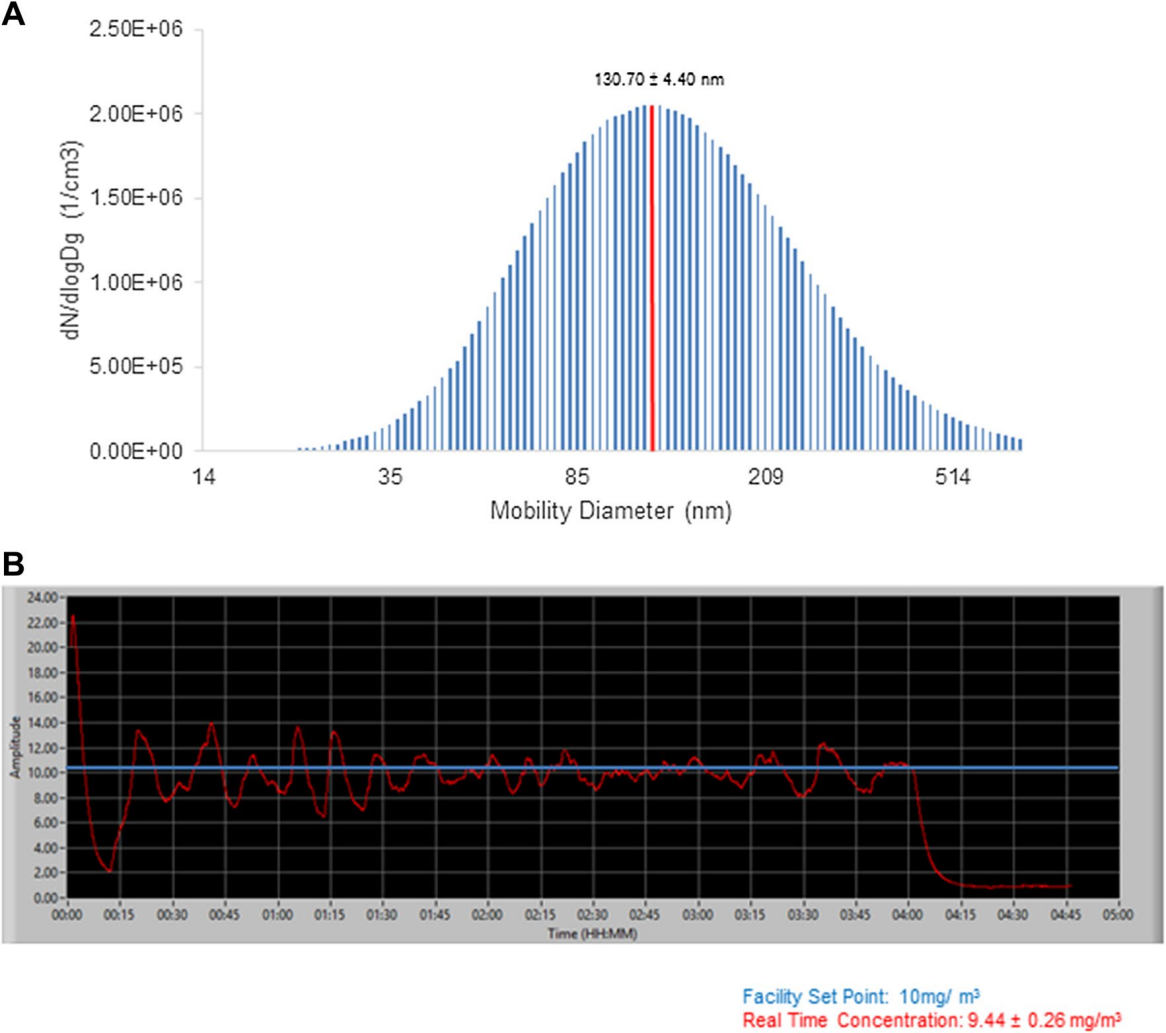
Exposure Characteristics. Real-time nano-TiO_2_ exposure characteristics. **a** Size-based aerodynamic diameter and mobility was characterized as 130.70 ± 4.40 nm. **b** A representative image of the real-time aerosol concentration of nano-TiO_2_ during the 4-h exposure, showing a maintained plateau at 9.44 ± 0.26 mg/m^3^. Values are means ± SE

Representative nano-TiO_2_ aerosol characterization data are presented in Fig. 2. The average median of our particle geometric size distribution was 130.70 ± 4.40 nm (Fig. 2a). A representative image of the real-time nano-TiO_2_ concentration of (9.44 ± 0.26 mg/m^3^) over the duration of the 4-h whole-body exposure demonstrates the ability of our custom inhalation exposure system to provide stable distribution of nanoparticles for acute applications (Fig. 2b). The calculated lung depositions of nano-TiO_2_ aerosols with consideration for clearance are presented in Table 1.

**Table 1:** Animal characteristics

Treatment	Maternal weight (g)	Mean arterial pressure	Number of rats (n)	Calculate particle deposition	Number of reabsorption sites	Number of pups	Pup weight (g)	Placenta weight (g)
Naive	380.92 ± 10.07		13.00	0	0.58 ± 0.23	13.08 ± 0.40	2.55 ± 0.02	0.43 ± 0.01
Air 4	366.60 ± 13.35	95.00 ± 2.77	5.00	0	0.20 ± 0.20	10.40 ± 2.44	2.56 ± 0.04	0.42 ± 0.01
Air 12	347.33 ±16.05	93.20 ± 2.54	6.00	0	0.00 ± 0.00	11.67 ± 1.65	2.48 ± 0.04	0.42 ± 0.01
Air 17	376.00 ± 16.05	92.75 ± 7.36	4.00	0	0.00 ± 0.00	12.50 ± 3.50	2.65 ± 0.03	0.45 ± 0.01
ENM 4	364.17 ± 11.78	96.80 ± 4.36	6.00	12.30 ± 0.34*	1.50 ± 0.76 (*p* = 0.15)	12.33 ± 0.56	2.50 ± 0.03	0.44 ± 0.01 (*p* = 0.08)
ENM 12	360.88 ± 11.43	84.75 ± 4.64	8.00	13.64 ± 0.37*	1.38 ± 0.18*	11.71 ± 0.64	2.65 ± 0.03*^†‡^	0.48 ± 0.01*^†^
ENM 17	378.71 ± 16.37	91.00 ± 3.78	7.00	15.79 ± 0.43*	0.71 ± 0.29*	12.14 ± 0.67	2.57 ± 0.03	0.49 ± 0.01^†^

Values are shown as mean ± SEM. Statistics were analyzed with a one-way analysis of variance (*p* ≤ 0.05)

*n* number of animals

*p ≤ 0.05 when compared with Air at each respective gestational day

^†^*p* ≤ 0.05 when compared with ENM 4

^‡^*p* ≤ 0.05 when compared with ENM 17

### Maternal, Litter, and Fetal Characteristics

Animal number, maternal body weight, MAP, number of reabsorption sites, litter size, pup weight, and placenta weight are reported in Table 1. At the time of the study, no statistically significant differences were observed between Naïve and Air groups. Further there were no statistically significant differences between groups with respect to maternal body weight, MAP, or litter size (Table 1). Number of reabsorption sites and placental weights were greater in animals exposed to TiO_2_ on GD 12 and 17 compared with animals exposed to filtered air on the equivalent GD. While the number of pups was not significantly different, those in the GD 12 exposure group were significantly larger than control, ENM 4, and ENM 17 counterparts.

### Vascular Responses in Naïve and Air Groups Following Nano-TiO_2_ Exposure

**Fig. 3:**
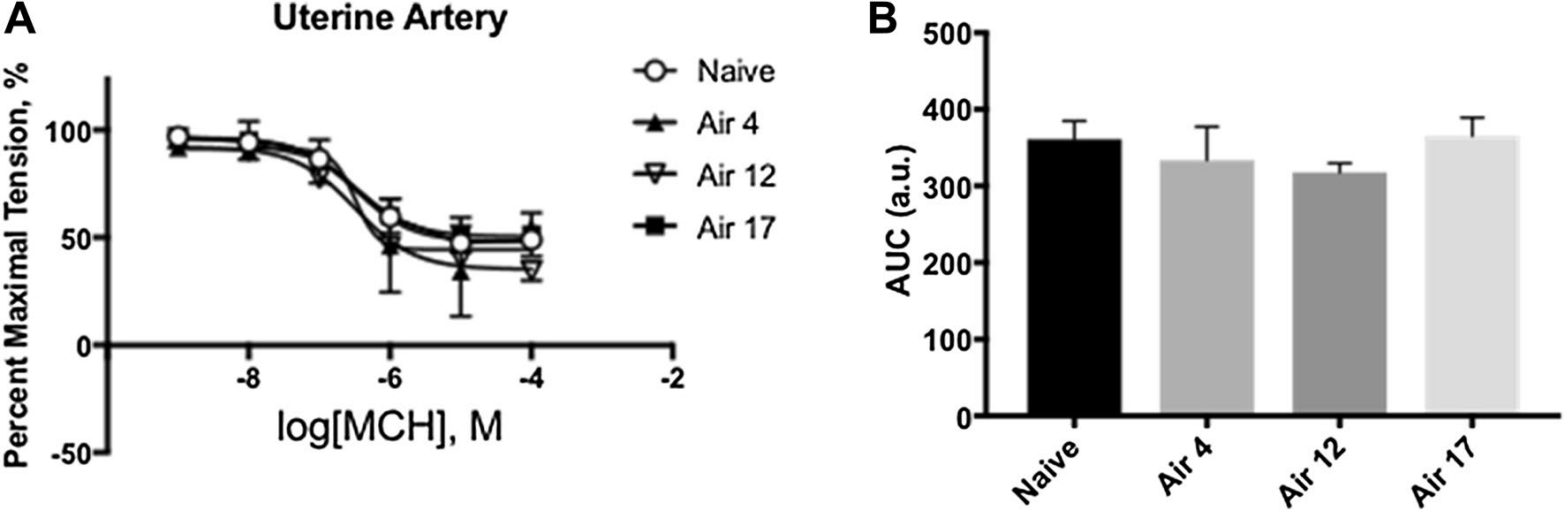
Representative endothelium-dependent relaxation in the uterine artery following a single exposure to filtered air at gestational day 4, 12, and 17 compared with naïve hemodynamic control. Percentage of maximum tension (**a**) and area under the reactivity curve (**b**) calculations are provided. No significant differences between groups. Therefore, we are confident pooling naïve and filtered air from different gestational day exposures (GD 4, 12, and 17) as representative “control”

There were no significant differences with respect to tension generation in response to PHE, or relaxation responses to MCH or SNP between Naïve and Air groups (data not shown). Data pertaining to endothelium-dependent function of the uterine artery are represented in Fig. 3a, b. Accordingly, cumulative response data for wire myograph investigations of vessel segments from Naïve and Air exposed groups were combined for simplification. When referencing the findings of the “control” group, we are referring to collective data from Naïve and Air groups.

### Changes in Vascular Responses Following Nano-TiO_2_ Exposure

#### Endothelium-Dependent Reactivity

**Fig. 4:**
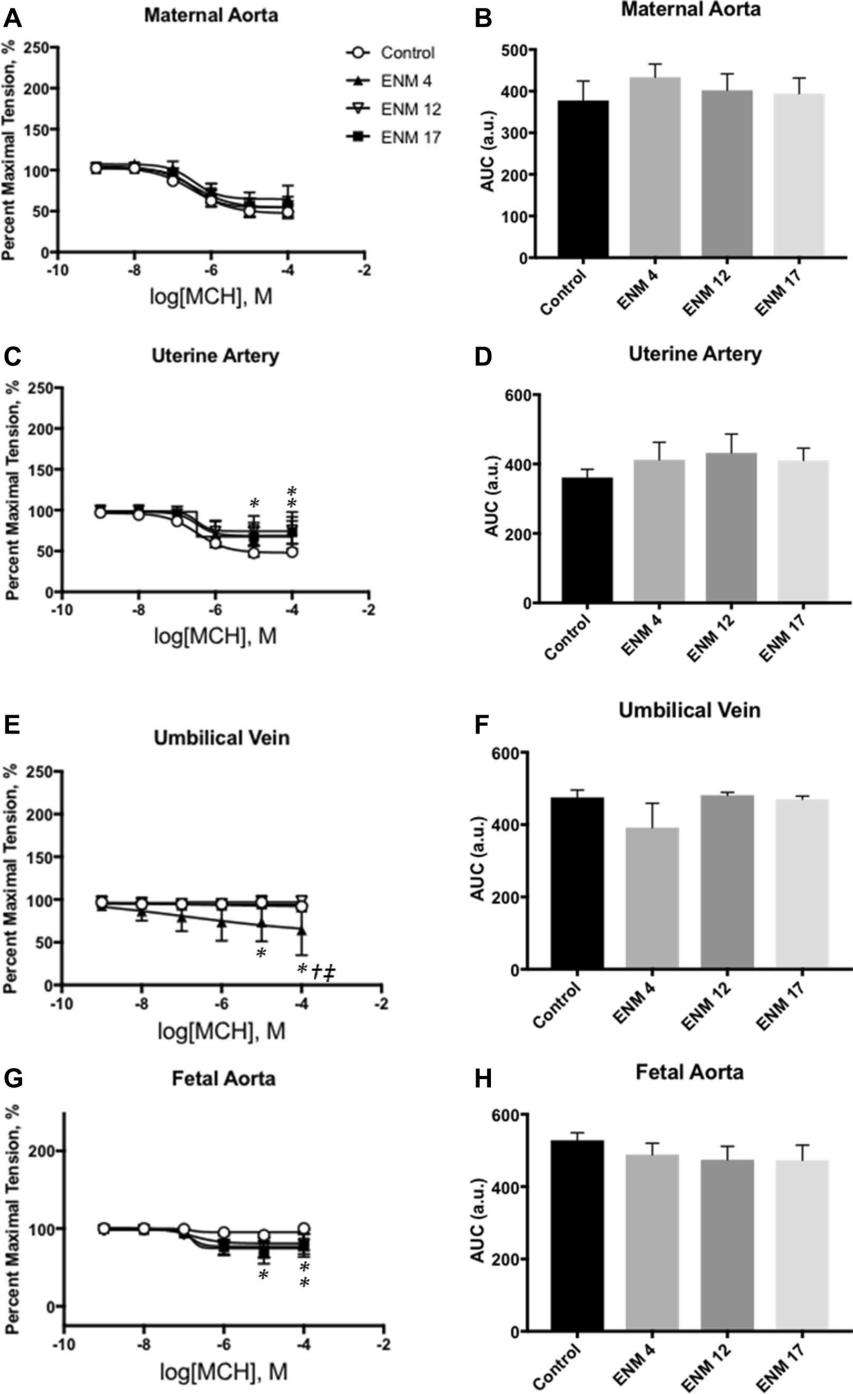
Changes in endothelium-dependent relaxation following a single exposure to aerosolized nano-TiO_2_ at gestational day 4, 12, and 17. The changes in the relaxation responses of segments of maternal and fetal vascular tissues at gestational day 20 in ENM-exposed and control Sprague Dawley rats in response to cumulative concentrations of methacholine (MCH) are provided for maternal thoracic aorta (**a**), uterine artery (**c**), umbilical vein (**e**), and fetal aorta (**g**), compared with maximum tension generation in response to KPSS. Area under the curve calculations are provided to represent overall segment reactivity of the maternal thoracic aorta (**b**), uterine artery (**d**), umbilical vein (**f**), and fetal aorta (**h**) to the MCH dose–response. *p ≤ 0.05 when compared with Control; ^†^*p* ≤ 0.05 when compared with ENM 12; ^‡^*p* ≤ 0.05 when compared with ENM 17. *n* = 6–13

No differences were reported in endothelium-dependent responses in maternal aortic segments between ENM and control groups (Fig. 4a, b).

Maximum relaxation responses from peak force within uterine artery segments derived from ENM 12 and ENM 17 animal exposures were significantly higher than control. Wherein preconstricted control artery segments relaxed by 48.90 ± 3.68%, 75.36 ± 22.53% for ENM 4, 75.29 ± 16.42% (*p* < 0.05 vs. control) for ENM 12, and 73.08 ± 13.89%; (*p* < 0.05 vs. control) for ENM 17 (Fig. 4c). However, overall uterine artery reactivity to increased concentrations of MCH was not significantly different between groups (Fig. 4d).

Fetal umbilical vein segments from animals exposed to ENM at GD 4 demonstrated significant relaxation at the highest concentrations of MCH (10^−5^ M–10^−4^ M) compared with control and later gestational ENM exposures (Fig. 4e). Analysis of the total response indicates an inability of the fetal umbilical vein to relax to cumulative concentrations of MCH (Fig. 4f). Physiologically, this could lead to reduced perfusion or higher blood pressures entering the fetal compartment.

Analysis of fetal thoracic aortic reactivity to discrete concentrations of MCH yields significance at the highest concentrations of MCH (10^−5^M–10^−4^M) (Fig. 4g). However, the overall relaxation response of fetal thoracic aortic segments to MCH was not different between ENM exposed and control (Fig. 4h).

#### Endothelium-Independent Reactivity

**Fig. 5:**
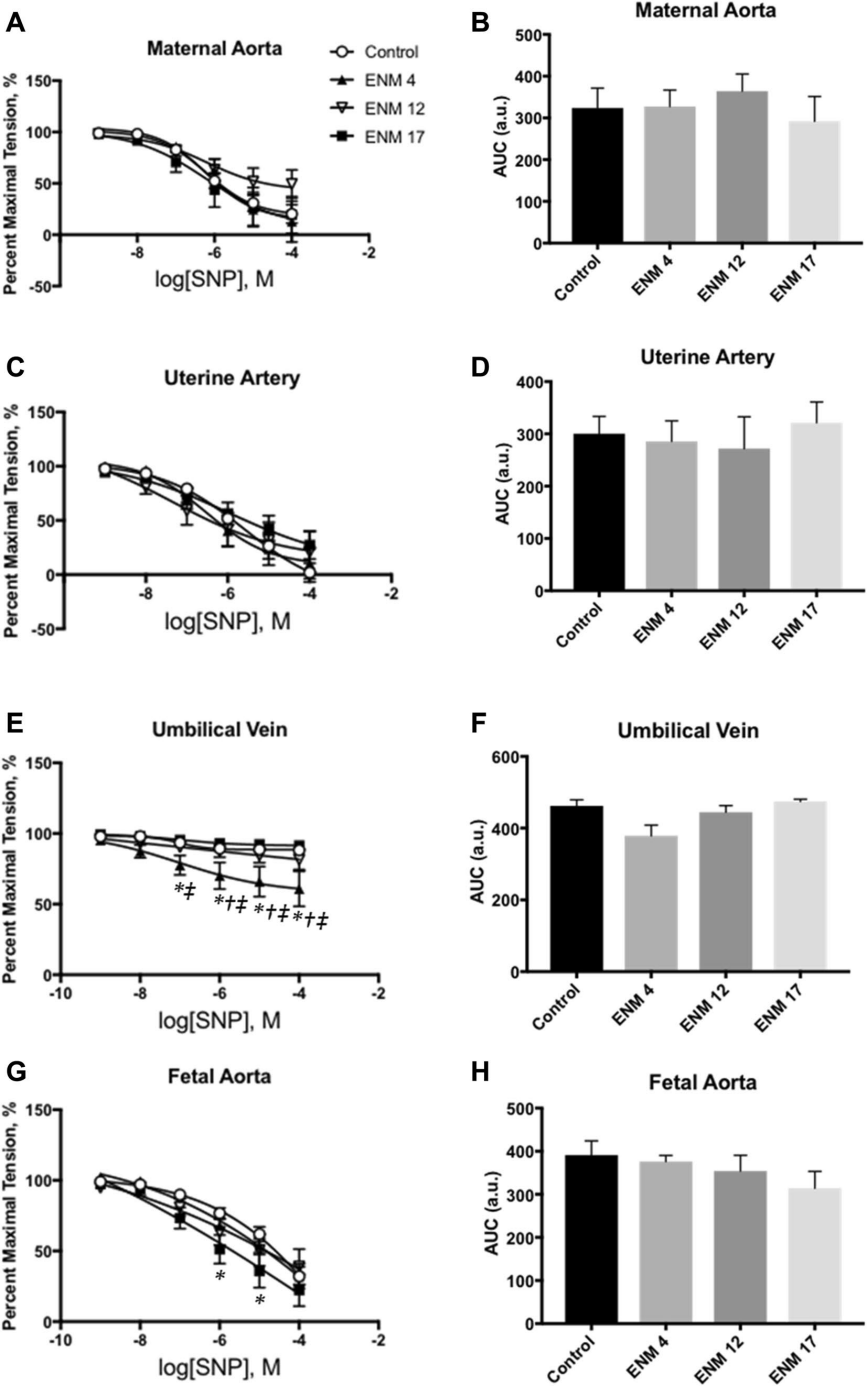
Changes in endothelium-independent relaxation following a single exposure to aerosolized nano-TiO_2_ at gestational day 4, 12, and 17. The changes in the relaxation responses of segments of maternal and fetal vascular tissues at gestational day 20 in ENM-exposed and control Sprague Dawley rats. Reactivity in response to cumulative concentrations of sodium nitroprusside (SNP) compared with maximum tension generation in response to KPSS is provided for maternal thoracic aorta (**a**), uterine artery (**c**), umbilical vein (**e**), and fetal aorta (**g**). Area under the curve calculations are provided to represent overall segment reactivity of the maternal thoracic aorta (**b**), uterine artery (**d**), umbilical vein (**f**), and fetal aorta (**h**) to the SNP dose–response. *p ≤ 0.05 when compared with Control; ^†^*p* ≤ 0.05 when compared with ENM 12; ^‡^*p* ≤ 0.05 when compared with ENM 17. *n* = 6–13

Endothelium-independent relaxation of the maternal aorta and the uterine artery, assessed in response to SNP, a spontaneous nitric oxide (NO) donor, after exposure to ENM at gestational day 4, 12, and 17 was consistent with responses from vessel segments in control animals suggesting a functional NO signaling cascade and subsequent smooth muscle relaxation (Fig. 5a–d).

Interestingly, point-by-point analysis revealed SNP induced significant concentration-dependent relaxation in umbilical vein segments following a single exposure at GD 4 (Fig. 5e). Umbilical vein segments derived from animals exposed to ENM on GD 4 were more responsive to SNP than either control or exposure groups at later gestational time points, indicated by a reduced area under the curve calculation; however, this value did not reach significance (Fig. 5f).

Umbilical Vein sensitivity to SNP was unaffected by single ENM exposure early in gestation (Fig. 5g). Endothelium-independent relaxation in response to SNP induced a significant relaxation in fetal thoracic aortic segments in ENM 17 at physiologically relevant concentrations (10^−6^M–10^−5^M). There was a trend toward an increase in sensitivity to SNP as assessed by area under the concentration response curve in ENM exposure from early to late gestation, but this did not reach statistical significance (Fig. 5h).

#### Smooth Muscle Contractility

**Fig. 6:**
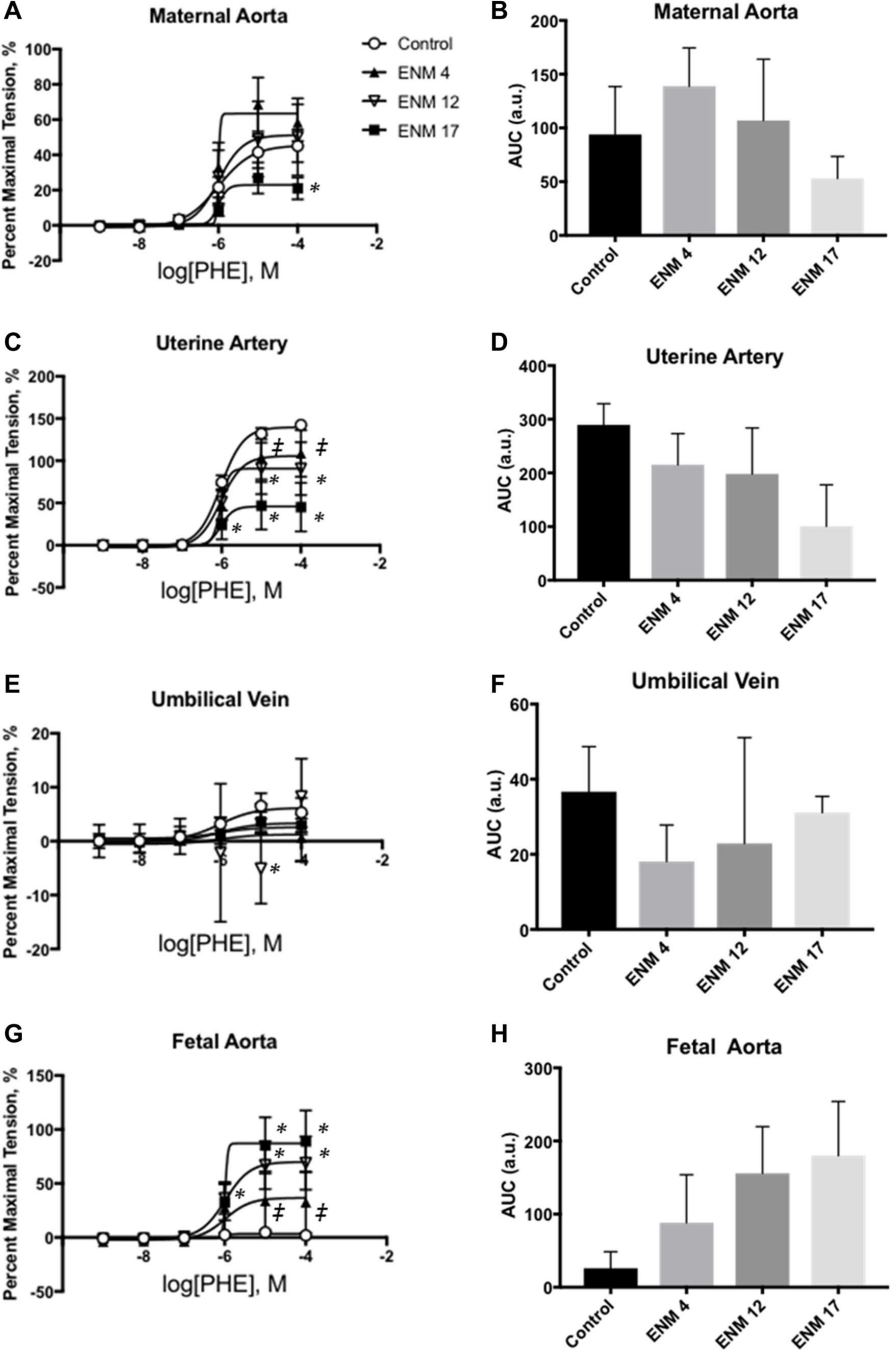
Changes in vascular smooth muscle contractility following a single exposure to aerosolized nano-TiO_2_ at gestational day 4, 12, and 17. The changes in tension generation of segments of maternal and fetal vascular tissues at gestational day 20 in ENM-exposed and control Sprague Dawley rats in response to cumulative concentrations of phenylephrine (PHE) are provided for maternal thoracic aorta (**a**), uterine artery (**c**), umbilical vein (**e**), and fetal aorta (**g**). Percent of maximal tension compared with KPSS responses is reported. Area under the curve calculations are provided to represent overall segment reactivity of the maternal thoracic aorta (**b**), uterine artery (**d**), umbilical vein (**f**), and fetal aorta (**h**) to the SNP dose–response. *p ≤ 0.05 when compared with Control; ^†^*p* ≤ 0.05 when compared with ENM 12; ^‡^*p* ≤ 0.05 when compared with ENM 17. *n*, number of animals. *n* = 6–13

Vascular smooth muscle contractility was assessed in response to the selective α-adrenergic receptor agonist PHE. Percent maximal tension generation in maternal aortic segments from animals exposed to nano-TiO_2_ on GD 17 was reduced by 24.0% in response to PHE when compared with control segments (Fig. 6a). However, sensitivity to PHE in animals exposed late in gestation (GD 17) was not significantly reduced overall when compared with control and animals exposed earlier in gestation. (Fig. 6b).

Smooth muscle contractility to maximal concentrations of PHE (10^−5^M–10^−4^M) was significantly diminished in uterine artery segments from ENM 12 (90.63 ± 31.27%) and ENM 17 (45.06 ± 28.85%) (Fig. 6c). However, these differences were not reflected in the overall hemodynamic responsiveness (Fig. 6d).

Significant differences were identified in the α-adrenergic contractility of the umbilical vein after maternal ENM inhalation on GD 12 of pregnancy. This was identified through a reduced contraction at supraphysiological concentrations (10^−5^M) (Fig. 6e).

α-adrenergic contractility to PHE (10^−^4M) was markedly increased in fetal thoracic aortic segments from ENM 12 (68.92 ± 24.73%) and ENM 17 (88.94 ± 28.61%) compared with control (1.93 ± 3.93%) (Fig. 6g). There was a trend toward an increase in sensitivity to PHE in fetal thoracic segments as assessed by area under the concentration response curve for ENM-exposed animals from early to late gestation, but due to variability within the responses, this did not reach significance (*p* = 0.8, 0.15, 0.068 compared with control for ENM 4, 12, 17, respectively) (Fig. 6h).

## Discussion

For this study, we assessed vascular reactivity in vessel segments from maternal, uterine, and fetoplacental circulations at vulnerable life stages. Alterations to vascular reactivity were identified on GD 20 after maternal exposure on GD 4, 12, and 17. Together, a single maternal exposure on GD 4 led to modification of vascular reactivity in each functional compartment (e.g., endothelium-dependent, -independent, and smooth muscle). Further, these variations were identified through serial assessments of the maternofetal circulation, with the greatest impairments identified in vascular smooth muscle reactivity, initiated through α-adrenergic signaling. Interestingly, fetal umbilical vein and fetal aorta reactivity were primarily modified in exposures initiated at GD 4. It is important to clarify that these single exposures took place 3, 8, and 16 days prior to assessment. Previous temporal studies of a non-pregnant occupational model identified epicardial arteriolar dysfunction for 7 days after multi-walled carbon nanotube inhalation [13]. Through these studies, we identified that a single maternal ENM inhalation exposure, even early in gestation, can have profound and long-lasting effects in maternal and fetal hemodynamic control.

Endothelium-dependent relaxation was assessed by generating cumulative concentration–response curves to MCH, following preconstriction with PHE. Reduced cholinergic activity was reported for uterine artery from animals exposed to ENM at early, mid, and late gestation. Earlier investigations by our group reported significantly blunted endothelium-dependent relaxation in isolated fetal tail arterioles following maternal exposure to nano-TiO_2_ aerosols [16]. Collectively, these findings in combination with our published work confirm maternal exposure to ENM during gestation impairs fetal systemic cardiovascular function. Of note, we reported poor agonist stimulated relaxation of vascular segments from umbilical vein and thoracic fetal aorta. These observations are consistent with existing literature [23] and indicate that cholinergic responses may not be the primary signaling pathway for endothelium-dependent dilation within the umbilical vein or fetal aorta. Poor reactivity may be due to reduced cholinergic receptor populations in these understudied tissue beds, reduction in NO production or bioavailability, or reliance on compensatory signaling mechanisms. Further assessments should consider alternate pharmacological agents to assess the contribution of nitric oxide production or the generation of metabolites of arachidonic acid (e.g., the nitric oxide synthase inhibitor L-N^G^-nitroarginine methyl ester and the cyclooxygenase antagonist indomethacin). In addition, future assessments may include examining the response to physiological modifications, including changes in shear stress and transmural pressure to further evaluate endothelial health.

Interestingly, fetal tissues derived after ENM 4 and ENM 17 exposures demonstrated an improved relaxation to NO indicating the possibility of increased NO-sensitivity after early/late gestation maternal ENM inhalation. These responses would further emphasize the consideration of alternate EDHF agonists in future work. The local production of reactive oxygen species has also been implicated in the development of contractile and bioenergetic dysfunction and impairments in endothelium-dependent vasoreactivity associated with exposure to nano-TiO_2_ [24, 25]. Accordingly, incubation with 2,2,6,6-tetramethylpiperidine-*N*-oxyl, a superoxide dismutase mimetic, and catalase, a hydrogen peroxide scavenger, would help to determine the influence of local reactive oxygen species production associated with nano-TiO_2_ exposure during pregnancy. Smooth muscle contractility was assessed in response to the selective α-adrenergic agonist, PHE. We observed that a single exposure to nano-TiO_2_ at mid (GD 12) and late (GD 17) gestation decreased maximum developed tension in the uterine artery. In contrast, smooth muscle contractility in the thoracic fetal aorta was significantly augmented in response to ENM exposure, an observation that was consistent at early, mid, and late gestation. Further, in ring segments from umbilical veins, contraction to 10^−2^M PHE was poor and variable. This observation is consistent with previous reports of agonist-induced contractile activity of murine umbilical arteries and veins [23]. In accordance with existing literature, exposure of umbilical vein segments to KPSS induced a maintained contraction. When compared with KPSS-induced contraction of uterine arteries, umbilical veins showed a significant decrease in maximal tension generation; similar findings have been reported in humans [26]. In isolated human umbilical vein rings, adrenaline was more potent than phenylephrine, 16% of the vessel rings did not demonstrate adrenergic responses to adrenaline or phenylephrine [27].

We observed differential responses between aortic, uterine, umbilical, and fetal aortic vessel segments with exposure to nano-TiO_2_ at multiple days across gestation. However, we have previously focused on the microcirculatory level of the vasculature [14, 16]. Given this context, our current work aligns with the findings of existing studies of serial vascular assessment [﻿28﻿], that while macrocirculatory reactivity is blunted, the greatest impairments are identified in the microcirculation. Overall, we postulate heterogeneous responses in vascular reactivity may be related to differences in cholinergic and α-adrenergic receptor populations and/or reliance on compensatory mechanism signaling cascades. This may include a shift in the role of arachidonic acid metabolites in the regulation of vascular tone (i.e., a disturbance in the ratio of the vascular smooth muscle dilator prostacyclin and the potent vasoconstrictor thromboxane A_2_) and/or increased reliance on Rho-kinase signaling [20, 22] after maternal ENM exposure.

Vascular remodeling during pregnancy, the time and pattern of which is specific to vessel size and location, is carefully orchestrated [29]. Pregnancy is accompanied by a progressive rise in uteroplacental blood flow (UPBF) in order to satisfy gestational demands of the developing fetus [1]. In rats, changes in total uterine blood flow are detected on or around the final third of gestation (~ GD 15) [30]. Increases in total UPBF are facilitated by a combination of circulatory adaptations including a rise in cardiac output and an expanded intravascular volume. Under normal circumstances, MAP decreases slightly or remains unchanged. Consistent with normal pregnancy, we observed no significant differences in MAP between naïve, sham control, and ENM-exposed groups. Accordingly, changes in cardiac output are accompanied by a reciprocal decrease in vascular resistance. Together, vessel-specific structural remodeling and a shift in uterine vascular reactivity (reduced vessel tone and enhanced vasodilation) determine pregnancy-related changes in UPBF [1]. Future studies may explore the influence of known pregnancy-related changes in the vascular micro-environment on vascular reactivity in pregnancy.

Technological advancements in nanomaterial science afford an abundance of opportunities for innovation. Nanotechnology has led to achievements in the accuracy and effectiveness of medical diagnostic equipment, vehicle fuel efficiency, sources of renewable energy, environmental remediation, and therapeutic efficiency of pharmaceuticals. While the unique physiochemical properties of engineered nanomaterials incite a wealth of enthusiasm, the pervasive impact of this technology also begets concerns that development may be outpacing our understanding of its safety. Exposure of the maternal environment to ENM during pregnancy has been shown to impact health of the dam, fetus, and adult offspring [31]; however, the consequences of specific temporal (gestational day) and spatial (vascular location) considerations are largely undetermined. In this respect, the current study is the first to examine how exposure to nano-TiO_2_ at critical periods of gestation affects vascular responses during pregnancy, and whether the resulting changes differentially affect regionally discrete blood vessels of maternal and fetal circulations.

In summary, the findings of this study suggest moderate vascular dysfunction associated with a single maternal exposure to nano-TiO_2_ aerosols. These results provide evidence that exposure early in gestation (GD 4) will have a significant impact on fetal systemic vascular function.

## Abbreviations

EDHF: Endothelium-derived hyperpolarizing factor
ENM: Engineered nanomaterials
GD: Gestational day
MAP: Mean arterial pressure
MCH: Methacholine
Nano-TiO_2_: Nano-titanium dioxide
PHE: Phenylephrine
PSS: Physiological saline solution
SNP: Sodium nitroprusside
UPBF: Uteroplacental blood flow
